# Association of Diabetic Neuropathy with Duration of Type 2 Diabetes and Glycemic Control

**DOI:** 10.7759/cureus.302

**Published:** 2015-08-12

**Authors:** Muhammad Umer Nisar, Ambreen Asad, Ahmed Waqas, Nazia Ali, Anam Nisar, Mohsin A Qayyum, Hafsa Maryam, Mohsin Javaid, Mohsin Jamil

**Affiliations:** 1 Final year MBBS Student, Yusra Medical & Dental College Near Kahuta Morr, GT Road, P.O Humak, Islamabad; 2 Associate Professor, Dept. of Physiology, Yusra Medical & Dental College Near Kahuta Morr, GT Road, P.O Humak, Islamabad; 3 Final year MBBS Student, CMH Lahore Medical College and Institute of Dentistry, Shami Road, Lahore Cantt.; 4 Fourth year MBBS Student, Yusra Medical & Dental College Near Kahuta Morr, GT Road, P.O Humak, Islamabad; 5 Wah Medical College, , POF Hospital Wah Cantt. Pakistan; 6 Fourth year MBBS Student , Yusra Medical & Dental College Near Kahuta Morr, GT Road, P.O Humak, Islamabad; 7 Fourth year MBBS, Yusra Medical & Dental College Near Kahuta Morr, GT Road, P.O Humak, Islamabad

**Keywords:** diabetes, diabetic neuropathy, peripheral neuropathy, diabetes mellitus, type 2 diabetes

## Abstract

Introduction: Diabetes mellitus is associated with severe microvascular and macrovascular complications with major implications for public health. Diabetic neuropathy is a very problematic complication of diabetes mellitus. It is associated with severe morbidity, mortality, and a huge economic burden. The present study was designed with two aims: 1) to analyze the association of diabetic neuropathy with the glycemic index (levels of fasting blood glucose, random blood glucose, and Hb1Ac) in patients with Type 2 diabetes, and 2) to analyze the association of diabetic neuropathy with time passed since the diagnosis of diabetes.

Methods: This case-control study was undertaken between June 2013 and February 2015 in the Armed Forces Institute of Rehabilitation Medicine (AFIRM), Rawalpindi, Pakistan. Type 2 diabetics with an age range of 30-60 years were recruited from outpatient departments of AFIRM, Rawalpindi. Data were collected and recorded on a form with four sections recording the following: 1) demographics of patients and number of years passed since diagnosis of diabetes; 2) clinical examination for touch, pressure, power, pain, vibration, and ankle reflex; 3) nerve conduction studies for motor components of the common peroneal nerve and tibial nerve and the sensory component of median nerve and sural nerve; 4) glycemic index, including fasting blood glucose levels (BSF), random blood glucose (BSR) levels, and HbA1c levels. Data were analyzed in SPSS v. 20. Chi-square and phi statistics and logistic regression analysis were run to analyze associations between diabetic neuropathy and time passed since diagnosis of diabetes and glycemic index.

Results: In total, 152 patients were recruited. One-half of those patients had neuropathy (76 patients) and the other half (76 patients) had normal nerve function. The mean (standard deviation [SD]) duration of diabetes was nine years (6.76), BSF levels 7.98 mmol/l (2.18), BSR 9.5 mmol/l (3.19), and HbA1c 6.5% (2.18). Logistic regression analysis predicted 87.5% of the model correctly. Duration since the diagnosis of diabetes and HbA1c levels were significantly associated with the diagnosis of neuropathy in diabetics.

Conclusion: The presence of diabetic neuropathy was significantly associated with HbA1c levels and the duration of diabetes.

## Introduction

In recent years, diabetes mellitus has become a serious public health concern in the developing world. In 2014, the nationwide prevalence of diabetes mellitus was estimated at 6.8% in Pakistan, with 87,548 diabetes-related deaths and a mean expenditure of $56 (USD) per person [1]. Therefore, diabetes poses a huge economic burden in Pakistan and this situation is projected to worsen with time.

Diabetes mellitus is a group of metabolic diseases associated with chronic hyperglycemia, which occurs as a consequence of destructive lesions of the pancreatic beta cells causing insufficient insulin secretion and several other etiological processes leading to decreased insulin sensitivity [2]. Subsequently, a plethora of metabolic derangements ensues that are further associated with several complications, such as retinopathy, neuropathy, nephropathy, macrovascular diseases [3], and depression [4]. These complications, if unchecked, may lead to potential blindness, foot ulcers, Charcot joints, amputations, and sexual dysfunction. In a low resourced country like Pakistan, about 3.5 million diabetics often go undiagnosed [1] and thus are at a higher risk of developing related complications.

Diabetic neuropathy is a very problematic complication of diabetes mellitus, associated with great morbidity, mortality, and a huge economic burden. Shera, et al. reported the prevalence of diabetic neuropathy to be 39.6% in Karachi [5]. Diabetic neuropathy consists of a family of neurological syndromes that affect specific regions of the nervous system, occurring in both Type 1 and Type 2 diabetes mellitus and also in acquired diabetes [6]. Its occurrence is explained by a multifactorial etiology that includes up-regulation of the polyol pathway, functional and structural microvascular disturbances, nervous and ganglionic hypoxia, increased oxidative stress, impairment in glycosylation of axonal and microvascular proteins, and impaired trophic factors required for peripheral nerves and their ganglia [8]. The San Antonio Convention divides neurological disturbances related to diabetes mellitus into subclinical neuropathy, assessed by anomalies in electrodiagnostic and quantitative sensory testing, and diffuse clinical neuropathy, involving distal sensorimotor and autonomic dysfunction and focal syndromes [7].

Neuropathy is associated with several risk factors, such as raised triglyceride levels, body mass index, smoking, hypertension [9], and diabetic microvascular [10] and macrovascular injuries [11]. Lack of insulin or C-peptide or both also promotes severe axonal atrophy and loss of axons [12]. Mismanagement of this complication can further lead to foot ulceration; a harbinger of gangrenous limb loss. According to the diabetic wound classification system proposed by Armstrong, et al., risk of amputation is increased by 1.7 times in the case of diabetic neuropathy, 12 times in case of deformity, and 36 times if there is a history of previous ulceration [13]. Progression of diabetes mellitus and associated complications can be controlled with good glycemic control by the patient. According to the Diabetes Control and Complications Trial Research Group, intensive therapeutic management of insulin-dependent diabetes reduces the onset and progression of diabetic complications by 35-70% [14]. There is overwhelming evidence that early insulin therapy provides neuroprotective effects in patients with Type 2 diabetes who have diminished insulin secretion [15].

The present study was designed with two aims: 1) to analyze the association of diabetic neuropathy with glycemic index (levels of fasting blood glucose, random blood glucose, and Hb1Ac) in patients with Type 2 diabetes, and 2) to analyze the association of diabetic neuropathy with time passed since diagnosis of diabetes.

## Materials and methods

This case-control study was undertaken between June 2013 and February 2015 in the Armed Forces Institute of Rehabilitation Medicine, Rawalpindi, Pakistan. Ethical approval was sought from the Ethical Review Committee of Yusra Medical and Dental College, Islamabad. One hundred and fifty-two patients with Type 2 diabetes were included in the study from the outpatient department of AFIRM, Rawalpindi. Due to limited resources, we could not ensure random sampling.

The minimum sample size required for this study was calculated to be 101 using the formula N = 10k/p where k is the number of covariates (k = 4), and p is the proportion of the smallest number of cases in the population (p = 0.39) [16].

Written consent was obtained from all patients following a comprehensive explanation of the purposes of the study. Inclusion criteria were: patients with previously diagnosed Type 2 diabetes and aged between 30-60 years with an intact site for testing for nerve conduction studies. Patients with serious illnesses or any other co-morbidities, musculoskeletal disorders, diagnosed or suspected neuropathy due to any other cause, and patients taking drugs that may have interfered with study results in any way were excluded from the study. After the medical history was recorded, the subjects underwent a detailed physical examination, which included checking the patient for vibration, touch, ankle jerk, and power of limbs by experienced physicians and authors, including MUN, MJ, MJ, NA, MAQ, and HM. A battery of tests, including blood glucose random (BSR), blood glucose fasting (BSF), and HbA1C, by pathologists in the hospital on an ADVIA-1800 apparatus and nerve conduction studies (NCS) by experienced clinicians on a MEDULAR DEVICE were conducted. Based on NCS results, the subjects were classified as either having a neuropathy or not. Those patients with diabetes with normal nerve function were taken as controls.

Data were collected and recorded on a pre-tested form with four sections: The first section assessed the number of years since diabetes was diagnosed. The respondents provided the data on the duration since diagnosis which could add recall bias. Therefore, the duration since diagnosis of diabetes was confirmed by reviewing the patient records to eliminate any recall bias. The second section recorded the data from the clinical examination for touch, pressure, power, pain, vibration, and ankle reflex. These were recorded as normal, decreased, or absent. The third section recorded NCS for motor components of the common peroneal nerve and tibial nerve as well as the sensory component of the median and sural nerves. Patients were recorded as normal if the NCS on all of the above nerves were normal, as having mild neuropathy if one sensory or one motor nerve conduction speed was abnormal or absent, as having moderate neuropathy if two or three nerve conduction speeds were abnormal or absent, and as severe if conduction abnormalities were present in all four nerves. The fourth section recorded the glycemic index of the recruited subjects, which included BSR, BSF and HbA1c levels.

Data were analyzed in SPSS v. 20. BSR, BSF, HbA1c, and duration since diagnosis of diabetes mellitus were dichotomized. These dichotomized variables had the following cut-off values: BSR (11.09 mmol/l), BSF (6.93 mmol/l), HbA1c (6.5%), and duration since diagnosis (three years). Results of the physical examination for touch, pressure, power, and ankle reflex were dichotomized as normal/abnormal. Chi-square and phi statistics were run to analyze the association of BSR levels, BSF levels, duration since diagnosis of diabetes, and HbA1c levels with the presence of neuropathy in patients with diabetes.

Then, these dichotomous variables (BSF, BSR, HbA1c levels, and duration since diagnosis) were introduced into a logistic regression model with a diagnosis of diabetic neuropathy (yes/no) as a dependent variable.

## Results

There were a total of 152 patients. Seventy-six patients (50.0%) had neuropathic conditions and 76 (50.0%) had normal nerve function. The mean (standard deviation [SD]) duration of diabetes was nine years (6.76), BSF levels 7.98 mmol/l (2.18), BSR 9.5 mmol/l (3.19), and HbA1c 6.5% (2.18). Detailed results of these variables with frequencies and Chi-square values are reported in Table 1. Chi-square analysis revealed a statistically significant, positive and moderate association between diabetic neuropathy and BSF levels greater than 6.9 mmol/l (φ = 0.337, P < 0.001), BSR levels greater than 11.1 mmol/l (φ = 0.493, P < 0.001), duration of diabetes greater than three years (φ = 0.456, P < 0.001), a strongly positive association of diabetic neuropathy with HbA1c levels > 6.5% (φ = 0.71, P < 0.001), and abnormal results of the physical examination for touch (φ = 0.74, P < 0.001), pressure (φ = 0.632, P < 0.001), power (φ = 0.782, P < 0.001), and ankle reflex (φ = 0.57, P < 0.001). Results of NCS tests also showed good association with diagnosis of neuropathy as assessed by the clinician.

**Table 1:** Frequency distribution of results, duration and diagnosis Frequency distribution of results of laboratory tests, duration since diagnosis, results of physical examination and clinical diagnosis in patients with diabetes with neuropathy and control group as assessed by NCS/EMG tests (n = 152)

Variable	Diabetics with Neuropathy (n =76)		Control Group (n =76)		P-value
	Frequency (n)	Percentage (%)	Frequency (n)	Percentage (%)	
Laboratory Tests					
BSR mmol/l					
≤ 11.1	37	34.3%	71	65.7%	< .001
>11.1	39	88.6%	5	11.4%	
BSF mmol/l					
≤ 6.9	17	28.8%	42	71.2%	< .001
> 6.9	59	63.4%	34	36.6%	
HbA1c (%)					
≤ 6.5%	14	17.1%	68	82.9%	< .001
> 6.5%	62	88.6%	8	11.4%	
Duration (years)					
≤ 3	4	10.5%	34	89.5%	< .001
> 3	72	63.2%	42	36.8%	
Physical Examination					
Power					
Normal	13	15.3%	72	84.7%	< .001
Abnormal	63	94%	4	6%	
Touch					
Normal	12	15%	68	85%	< .001
Abnormal	64	88.9%	8	11.1%	
Ankle reflex					
Normal	33	31.1%	73	68.9%	< .001
Abnormal	43	93.5%	3	6.5%	
Pressure					
Normal	26	26.5%	72	73.5%	< .001
Abnormal	50	92.6%	4	7.4%	
Clinical Diagnosis					
Normal	1	1.5%	65	98.5%	< .001
Neuropathic	75	87.2%	11	12.8%	

A logistic regression was performed to ascertain the effects of BSR, BSF, HbA1c levels, and duration since diagnosis of diabetes on the likelihood that participants have diabetic neuropathy. These covariates predicted 87.5% of the model correctly. Out of four predictor variables, only two were statistically significant: HbA1c and duration since diagnosis of diabetes (Table 2). Diabetics with duration of more than three years since diagnosis of diabetes were 7.8 times more likely and diabetics with HbA1c levels greater than 6.5% were 16.9 times more likely to develop neuropathy. HbA1c levels were the strongest predictor of diabetic neuropathy.

**Table 2:** Binary logistic regression model with nerve function status (normal/neuropathic) as dependent variable (n=152) Cox & Snell R Square = 0.491, Negelkerke R Square = 0.654; Hosmer & Lemeshow Test: P = 0.102, Model P value < 0.001

Variables	B	P value	Odds Ratio	Lower CI	Upper CI
Random blood sugar levels (mmol/l)					
≤ 11.1	1.3	0.048	1		
>11.1			3.7	1.01	13.34
Fasting blood sugar levels (mmol/l)					
≤ 6.9	0.37	0.487	1		
> 6.9			1.4	0.51	4.06
HbA1c levels (%)					
≤ 6.5	2.83	< 0.001	1		
> 6.5			16.9	5.71	50.10
Duration (years)					
≤ 3	2.1	0.003	1		
> 3			7.8	1.99	31.81
Constant	-3.4	< 0.001	0.032		

According to the logistic regression analysis, diabetic neuropathy exhibited a weak and marginally significant association with BSR levels (P = .048). Diabetics with BSR levels > 11.1 mmol/l had 3.7 times (95% CI= 1.01 - 13.34) higher odds to exhibit neuropathy. 

## Discussion

Diabetic neuropathy is a severe complication of diabetes, and it is associated with multiple risk factors. This study evaluated the association of duration of diabetes mellitus and glycemic control with diabetic peripheral neuropathy. According to our analysis, both poor glycemic control and longer duration were significantly associated with diabetic neuropathy.

A long duration of diabetes and poor glycemic control is associated with increased production of glycosylation end products, metabolic derangements, endothelial injury, and oxidative products [17-18]. Oguejiofor, et al. found a lower prevalence of polyneuropathy in those with duration of DM < 5 years and highest in those with a duration of DM > 15 years [19]. A large study in the UK showed that neuropathy was present in as many as 36% people with duration of diabetes greater than 10 years as compared to 20% when duration of diabetes was five years [20]. Sensory neuropathy and the extent of skin denervation also increases with duration of diabetes [21]. The association between the duration of diabetes mellitus and neuropathy was also evident in a research study on the epidemiology of diabetic complications [22].

In the present study, a logistic regression model consisting of duration of diabetes, HbA1c, BSF, and BSR predicted 87.5% of the occurrence of diabetic neuropathy in the study sample. Patients with an HbA1c > 6.5% were 16.9 times more likely to develop neuropathy. The role of poor glycemic control and chronic hyperglycemia as a risk factor for diabetic neuropathy has also been established in several longitudinal studies [23-24]. The severity of hyperglycemia and abnormal glycemic hemoglobin levels considerably affect the results of the sensory and motor NCS tests [25]. This might be due to the fact that abnormal levels of HbA1c are positively associated with neuromuscular jitters and fiber densities [26]. These variations in HbA1c are also associated with other diabetic complications as evident in a multicenter study, which has established variation in HbA1C to be an important risk factor of diabetic retinopathy [27]. Tight blood glucose control significantly reduced the risk of microvascular complications in the Diabetes Control and Complications Trial (DCCT), which showed that intensive insulin therapy reduced incidence of albuminuria by 54% and decreased mean risk of retinopathy by 76% [14]. The United Kingdom Prospective Diabetes Study (UKPDS) showed that the intense glucose control group had a 25% reduction in the risk of microvascular complications endpoints. Furthermore, UKPDS showed that keeping HbA1c at a mean of 7% over 10 years significantly reduced the risk of microvascular complications [28]. These studies also recommend a target HbA1c as close to normal as possible, which provides improved outcomes [14, 29]. The UKPDS showed that HbA1c below 6% had the lowest risk for diabetes-related complications and for every 1% decrease in the mean HbA1c, there was a 37% decrease in microvascular complications [29]. Patients in the UKPDS who were intensively treated for diabetes and maintained an HbA1c below 7% had a 12% decrease in diabetes-related microvascular events. The Action in Diabetes and Vascular Disease (ADVANCE) trial showed that after a five-year follow-up in an intensively treated group with mean HbA1c of 6.5% as compared to a standard treated group with mean HbA1c of 7.3%, there was an decreased incidence of microvascular events (9.4% vs. 10.9%; hazard ratio: 0.86), especially because of the decreased incidence of nephropathy (4.1% vs. 5.2%; hazard ratio, 0.79) [30]. Glycemic control significantly affects the rate of progression from microalbuminuria to proteinuria and from overt nephropathy to ESRD [31]. A prospective study in Japan divided patients into two groups: multiple insulin injections group (MIT) and conventional insulin injection group (CIT). This study showed a decreased risk of nephropathy and retinopathy and an improvement in neurological tests like NCS in the MIT group while the CIT group showed deterioration in nerve conduction velocities and vibration threshold. The study concluded that keeping HbA1c at about 6.5% by multiple insulin injection therapy can delay the onset and the progression of diabetic microvascular events [32]. DCCT showed that, with a median HbA1c of 7%, there is 35–76% reduction in the early stages of microvascular disease [33]. Tight glycemic control is required to prevent microvascular and macrovascular complications, which is particularly effective in the early phase of disease [34]. These studies show tight glycemic control, which is measured by HbA1c as the most important factor to decrease microvascular events. Diabetic neuropathy is also associated with several other risk factors, including age, smoking, serum cholesterol levels, cardiovascular disease, increased diastolic blood pressure, severe ketoacidosis, raised levels of fasting triglyceride, proliferative diabetic retinopathy, and increase in urine albumin [35]. Intensive multifactorial long-term interventions have been proved to slow progression of auto autonomic neuropathy, nephropathy, and retinopathy and reduce the risk of cardiovascular disease [36-37].

There is overwhelming evidence that timely screening with earlier detection and intervention would be useful in preventing the progression of neuropathy and reduced risk of complication in other organs [38-39]. Effective communication between healthcare professionals and patients with diabetes can greatly reduce the morbidity and mortality associated with diabetes. This has also been highlighted by Mahdad, et al. who found compelling evidence of improvement in glycemic control, redox, and inflammatory status of diabetic patients after a three-month lifestyle advice follow-up [40]. After a rigorous literature review, Basit, et al. devised “BRIGHT” guidelines for self-monitoring of blood glucose levels, which highly individualizes the therapeutic regimen for patients with diabetes by accurate assessment of metabolic control, forming realistic goals, and preventing chronic complications and cognitive decline [41]. To increase compliance in Pakistani diabetic patients, their attitudes, concerns and fears regarding the etiology, progression, efficacy, and side effects of insulin and oral hypogylcemics should be properly addressed by sharing information adequate to their literacy levels and culture.

## Conclusions

Diabetic neuropathy is a very problematic complication of diabetes. It is significantly associated with HbA1c levels and duration of diabetes.
